# Corosolic acid isolated from *Eriobotrya japonica* leaves reduces glucose level in human hepatocellular carcinoma cells, zebrafish and rats

**DOI:** 10.1038/s41598-019-40934-7

**Published:** 2019-03-13

**Authors:** Shuwen Xu, Gang Wang, Wei Peng, Yandi Xu, Yu Zhang, Ying Ge, Yue Jing, Zhunan Gong

**Affiliations:** 1 Center for New Drug Research and Development, College of Life Science, Nanjing Normal University, Nanjing, 210023 People’s Republic of China; 2 Department of Scientific Research Management, Anhui Academy of Science and Technology, Hefei, 230088 People’s Republic of China; 3 Central Laboratory of Stomatology, Nanjing Stomatological Hospital, Medical School of Nanjing University, Nanjing, 210008 People’s Republic of China

## Abstract

Type 2 diabetes (T2D) with high morbidity and mortality is characterized by abnormal glucose and lipid metabolism due in part to insulin resistance in liver, which lead to elevated hyperglycemia and hyperlipidemia. This study sough to explore the effects of corosolic acid (CA) in different T2D models and explored the underlying mechanism. Separated from *Eriobotrya japonica* leaves, CA purity was above 95% measured by a HPLC method. Compared with cAMP and DEX induced T2D HepG2 model, CA significantly stimulated glucose consumption and improved glycogen accumulation by inhibiting *PEPCK* mRNA expression. And in cAMP and DEX induced T2D zebrafish model, CA reduced glycogen degradation and increased glucose consumption by regulating some key enzymes in carbon metabolism including GLUT1, GLUT2, GLUT3, LDHA, LDHB, GP, G6Pase, GYS1, and PFKFB3. In addition, insulin receptor signals were also involved in CA-regulated hypoglycemic action. Furthermore, in STZ-induced T2D rat model, compared with diabetic control groups, CA remarkably downregulated the levels of serum lipid, blood glucose, ICAM-1, malonaldehyde and insulin resistance index, while upregulated SOD activity and impaired glucose tolerance. In a conclusion, CA can regulate glucose and lipid metabolic adaptation in T2D like HepG2, zebrafish and rat models partly through reducing inflammation and oxidative stress and suppressing *PEPCK*.

## Introduction

Type 2 diabetes(T2D) is a widely heterogeneous disorder featured by fasting and post-prandial hyperglycemia and progression of insulin resistance, which has affected increasing number of populations and become one of the most costly and burdensome diseases^1^. Despite great efforts have been made to normalize blood glucose level in clinic therapy, it’s still a formidable challenge^2^.

It is essential to maintain blood glucose levels in a normal range for the health of human body, because hyperglycemia can cause damage to protein dysfunction and oxidative stress led by nonspecific glycosylation. Excessive hepatic glucose output is the main cause of hyperglycemia in diabetic patients. Therefore, alleviating excessive hepatic gluconeogenesis is a potential target for the treatment of glucose metabolic disorders^3^. Phosphoenolpyruvate carboxykinase (PEPCK) is a rate-limiting enzyme in liver and kidney gluconeogenesis, which catalyzes the conversion of oxaloacetate (OAA) into phosphoenolpyruvate (PEP)^4^. The transcription of PEPCK can be regulated by hormones involved in glucose homeostasis, for example, glucagon (acting by the second messenger cAMP), glucocorticoid (dexamethasone(DEX)) and retinoic acid^5^, dominantly repressed by insulin^6^. The overexpression of PEPCK has been found in almost all diabetes models, therefore, PEPCK can be used as an indicator of blood glucose levels^7,8^.

At present, many therapeutic agents for T2D treatment, like sulphonylures and bisguanidine, are responsible for side effects like flatulence and diarrhea^9^. Thus, much attention has been paid to active natural products from Chinese herbal medicines, which have the functions on controlling glucose level and alleviating the risk of complications^10^.

It’s previously reported that terpenoids are strikingly lower-blood glucose compounds by enhancing insulin sensitivity, promoting insulin secretion, inhibiting protein tyrosine phosphatase 1B, α-glucosidase, aldose reductase (AR) and dipeptidyl peptidase-4(DPP-4) activities to further reducing liver glycogen decomposition and gluconeogenesis^11^. Corosolic acid (2α,3β-2,3-dihydroxy-urs-12-en-28-oic acid) is a natural pentacyclic triterpene discovered in traditional Asian medical herbs such as *Lagerstroemia speciose*^12,13^, *Eriobotrta japonica*^14^, *Tiarella polyphylla*^15^, which has attracted much attention for its antidiabetic and anti-lipid oxidative activity in some animal experiments and clinical trials, including development of glucose metabolism by declining insulin resistance in mice and reducing on post-challenge plasma glucose levels in human^16–25^. Although corosolic acid was reported to be a talented compound for diabetes, its underlying mechanism has not been systematically analyzed.

As a vertebrate, due to high homology with human, zebrafish has been widely used as a model organism to research on biological process of vertebrate. These studies include malignant neoplasms, cardiovascular diseases, immune system diseases, antioxidant stress and diabetes^26^. It’s been reported that zebrafish regulated glucose metabolism by producing insulin, glucagon and other important proteins, such as PEPCK, which identified similar regulation patterns and activity in mammalian models^27,28^.

In our study, we firstly separated corosolic acid from *Eriobotrya japonica* leaves, investigated the effect of corosolic acid on glucose and lipid metabolism and identified the molecular mechanism of corosolic acid alleviated hyperglycemia in different T2D models containing cAMP and DEX-induced HepG2 cells, cAMP and DEX-induced zebrafish and STZ-induced rats.

## Results

### Separation and identification of CA

After dried and powdered, *Eriobotrya Japonica* leaves were extracted by ethanol and concentrated to get the extract. Discard the supernatant, dissolve the residue by ethanol again, and decolor it by activated carbon repeatedly. After filtration, the supernatant could be reduced pressure concentrated and dried to prepare the crude extract. Dissolve the crude extract by methanol and filtrate it with 0.45μm membrane in order to do preparative HPLC separation. In contrast to CA standard, the corresponding peak effluent of CA was collected, combined, concentrated under reduced pressure, and finally recrystallized with methanol to get corosolic acid.

**Figure 1:**
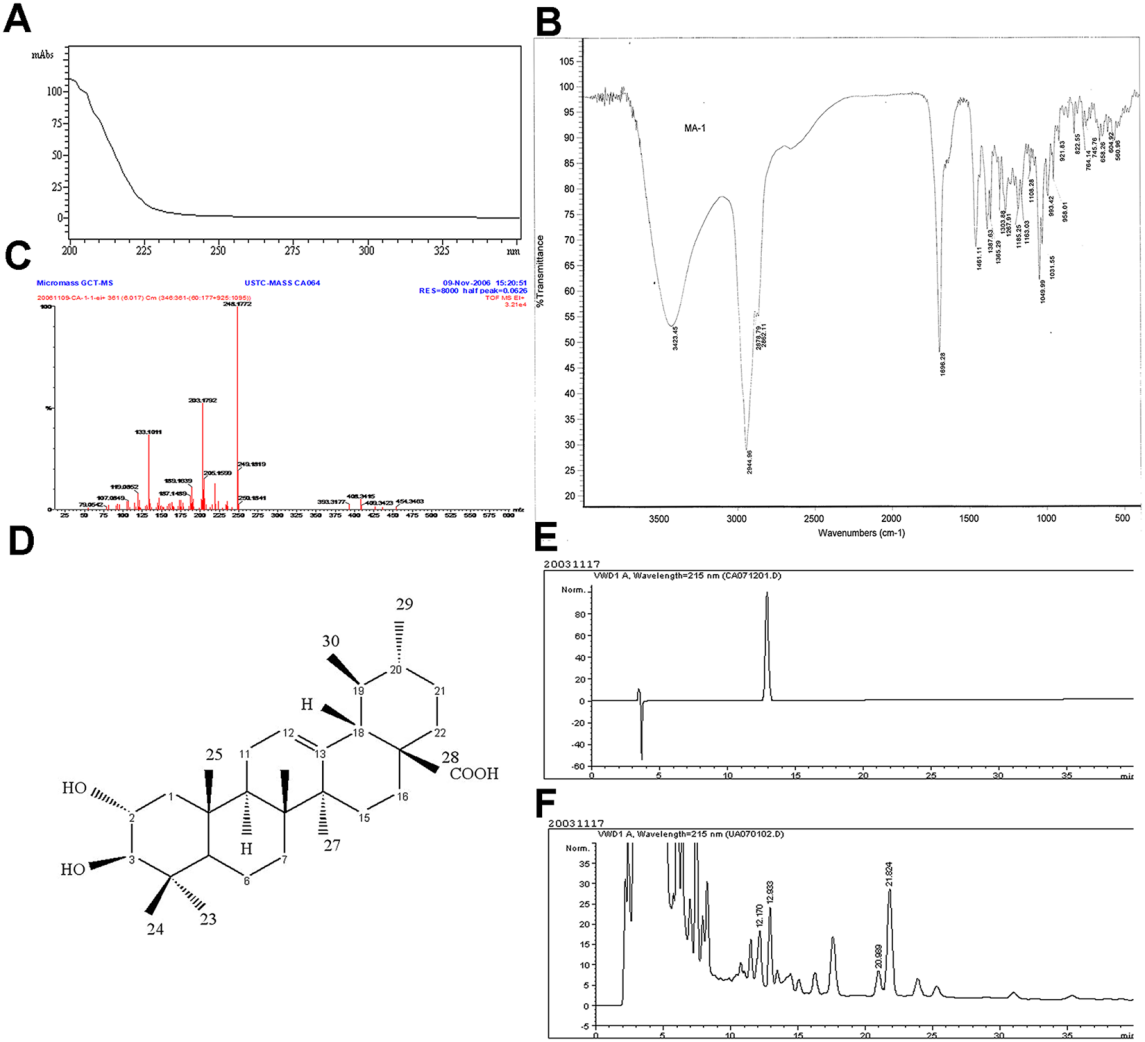
Separation, identification and determination of CA. (**A**) UV analysis for products separated by preparative chromatography; (**B**) IR spectra of CA; (**C**) EI-MS spectra of CA; (**D**) Chemical structure of CA; (**E**) HPLC chromatograms of standard CA; (**F**) HPLC chromatograms of four triterpene acids separated from *Eriobotrya Japonica* leaves.

Since the target sample structure contains only chromophores such as saturated alkanes, cyclohexene, mono-carboxyl and hydroxyl groups, simultaneously the maximum absorption wavelength of these chromophores all fall within the deadline wavelength of methanol, only the terminal absorption can be seen in the UV-visible spectrum (Fig. 1A).

Samples were plated with KBr. The IR absorption bands at 3415, 2973, 2927, and 2872 cm^−1^ indicated the presence of hydroxy, carbonyl and alkene (Fig. 1B).

Corosolic acid isolated as a white powder, exhibited an EI-MS ion at m/z 454.3463[M-18]^+^ (calcd for C_30_H_46_O_3_, 454.3447) (Fig. 1C) and combined with the ^13^C NMR data (Table 1), displayed 30 carbon resonances, which were classified as 7 methyl, 8 methylene, 8 methine, and 7 quaternary, which suggested a molecular formula of C_30_H_48_O_4_ (Fig. 1D). Its ^1^H NMR data (Table 1) showed signals for 7 methyl groups at δ H0.71 (3 H, s, H29), 0.75(3 H, s, H30), 0.82(3 H, s, H25), 0.92(3 H, s, H24), 0.92(3 H, s, H26), 0.92 (3 H, s, H27), 1.0(3 H, s, H23), an olefinic proton at δ 5.17(1 H, t, J = 3.4 Hz, H12), and a tertiary carbon proton at δ 2.11 (1 H, t, H20).

**Table 1:** ^13^C NMR data (500 MHz, DMSO-*d*_6_).

position	δ_C,_ type	position	δ_C,_ type
1	46.8,CH_2_	16	23.7,CH_2_
2	67.1,CH	17	46.9,C
3	82.2,CH	18	52.3,CH
4	38.8,C	19	38.4,CH
5	54.7,CH	20	38.4,CH
6	18.0,CH_2_	21	30.1,CH_2_
7	32.6,CH_2_	22	36.2,CH_2_
8	39.0,C	23	28.8,CH_3_
9	47.0,CH	24	17.1,CH_3_
10	37.5,C	25	16.4,CH_3_
11	22.9,CH_2_	26	16.9,CH_3_
12	124.4,CH	27	23.2,CH_3_
13	138.2,C	28	178.2,C
14	41.6,C	29	21.0,CH_3_
15	27.4,CH_2_	30	16.9,CH_3_

#### Corosolic acid

white powder; mp254–256 °C; IR (Nujol) 2973, 2927, and 2872 cm^−1^; ^1^H-NMR(DMSO-d_6_, 500 MHz) δ: 0.71(3 H, s, H29), 0.75(3 H, s, H30), 0.82(3 H, s, H25), 0.92(3 H, s, H24), 0.92(3 H, s, H26), 0.92(3 H, s, H27), 1.0(3 H, s, H23), 4.22(1 H, d, J = 8.7 Hz, H-2β), 4.33(1 H, dd, J = 9.0, 11.5, H-3α), 5.17 (1 H, t, J = 3.4 Hz, H12), 3.42(1 H, t, J = 9.8 H18), 2.74(1 H, t, H19), 2.11 (1 H, t, H20);^13^C-NMR(DMSO-d_6_,125 MHz)δ178.2(COOH, C-28), 138.2(C, C-13), 124.4(CH, C-12), 82.2(CH, C-3), 67.1(CH, C-2), 54.7(CH, C-5), 52.3(CH, C-18), 47(CH, C-9), 46.9(C, C-17), 46.8(CH_2_, C-1), 41.6(C, C-14), 39(C, C-8), 38.8(C, C-4), 38.4(CH, C-19), 38.4(CH, C-20), 37.5(C, C-10), 36.2(CH_2_, C-22), 32.6(CH_2_, C-7), 30.1(CH_2_, C-21), 28.8(CH_3_, C-23), 27.4(CH_2_, C-15), 23.7(CH_2_, C-16), 23.2(CH_3_, C-27), 22.9(CH_2_, C-11), 21(CH_3_, C-29), 18(CH_2_, C-6), 17.1(CH_3_, C-24), 16.9(CH_3_, C-26), 16.9(CH_3_, C-30), 16.4(CH_3_, C-25).

Furthermore, comparing to CA standard(Fig. 1E), a HPLC-UV method was built to detect purity of CA (Fig. 1F) by determining the peak with S/N greater than 3 as impurities (>96.1%, shown in Supporting Information, S1), which was also confirmed by melting range of 254–256 °C, the same as related references^29^. In addition, the average content of CA in *Eriobotrya Japonica* leaves is 0.814% (Table 2).

**Table 2:** Determination of CA content in *Eriobotrya Japonica* leaves.

Number	CA Content/(mg/g)
110106	8.66
110307	7.88
110510	7.91
110804	8.13
111108	8.22
average	8.14

### CA stimulated glucose consumption and improved glycogen accumulation by regulating PEPCK in HepG2 cells

**Figure 2:**
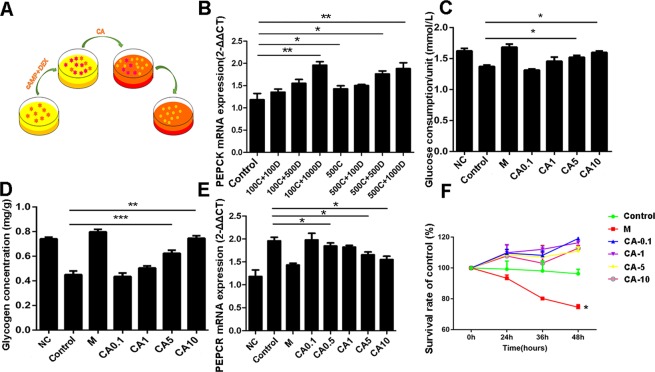
CA improved glucose consumption and glycogen stimulate led by cAMP and DEX induced T2D cell model. (**A**) Model building schematic diagram; (**B**) PEPCK mRNA expression in different cAMP and DEX treated group; After CA treatment, glucose consumption (**C**), glycogen stimulate (**D**) and PEPCK mRNA expression (**E**) were analyzed; (**F**) Antiproliferative effect of CA on HepG2 cells were analyzed by CCK8. * Indicates a significant difference from the control at *P* < 0.05, **P < 0.01, ***P < 0.0001. C, cAMP; D, DEX; M, metformin.

Phosphoenolpyruvate carboxykinase (PEPCK) is a key rate-limiting enzyme of gluconeogenesis in the liver^30^. The expression of PEPCK is regulated at transcriptional level and regulated by glucose-related hormones involved in glucose homeostasis, such as glucagon, glucocorticoid and insulin^31^. So far, overexpression of PEPCK has been found in many diabetes models and induction of PEPCK overexpression in rodent models can induce insulin-resistant, a kind of Type 2 diabetes (T2D) like syndrome^6^. In our study, we established type 2 diabetic-like model in HepG2(details in Supporting Information. S2, S3, Fig. S1, S2) stimulated by cAMP and DEX (Fig. 2A). After testing the effect and stability of insulin-resistant hepatocytes, the expression of PEPCK was further detected. The result in Fig. 2B showed that PEPCK expressions in different treatments of cAMP and DEX were higher than that in the control group. Especially, PEPCK expressions in groups of 100 μM cAMP + 1000 nM DEX, 500 μM cAMP, 500 μM cAMP + 500 nM DEX, and 500 μM cAMP + 1000 nM DEX were much higher than control with significant difference. Therefore, the expression level of PEPCK in HepG2 cells treated with 100 μM cAMP + 1000 nM DEX (*P* < 0.01), which was our induced condition of T2D model, was obviously higher than control in accordance with glucose consumption and glycogen accumulate.

Next, we analyzed glucose consumption and glycogen stimulate after treating by CA in insulin-resistant hepatocytes. In Fig. 2C,D, the unit glucose consumption and intracellular glycogen content of CA treated groups upregulated with the increasing concentration of CA. The unit glucose consumptions of 5 μM and 10 μM CA treated group were 1.11 times and 1.16 times higher than cAMP and DEX treated group, respectively (*P* < 0.05). In addition, 5 μM and 10 μM CA could significantly improve the intracellular glycogen degradation induced by cAMP and DEX, and 10 μM CA regulated glycogen content change was almost no significant difference with metformin treated group (*P* < 0.01). To sum up, 10 μM CA can significantly improve the decrease of glucose consumption and intracellular glycogen content induced by cAMP and DEX, maintain glucose homeostasis and reduce glucose toxicity caused by excess glucose. To investigate the mechanism of hypoglycemic activity of CA in HepG2 cells, the expression of PEPCK was evaluated in mRNA level, which is a well-known enzyme involved in type 2 diabetes. As shown in Fig. 2E, the expression of PEPCK was decreased by CA treatments, and significant differences were observed at 5 μM and 10 μM concentration(*P* < 0.05). It was worth that the effect of 10 μM CA on reversing the overexpression of PEPCK in T2D model was nearly similar to that of metformin.

In addition, we further detected the toxicity of CA on HepG2 cell at different concentrations. The survival rate in Fig. 2F presented CA showed no significant effect on cell viability in the concentration range of hypoglycemic action, even if at maximum concentration of 10 μM.

### CA reduced glycogen output by regulation of carbon metabolism and insulin receptor in zebrafish model

It has been reported that zebrafish have great potential in the study of diabetes. Exposure of zebrafish to a high concentration of glucose solution can induce metabolic changes similar to hyperinsulinemia and damage glucose metabolism in peripheral tissues. And it also been found that adult zebrafish respond to diabetes drugs by lowering blood sugar levels, which is similar to that in mammalian models.

**Figure 3:**
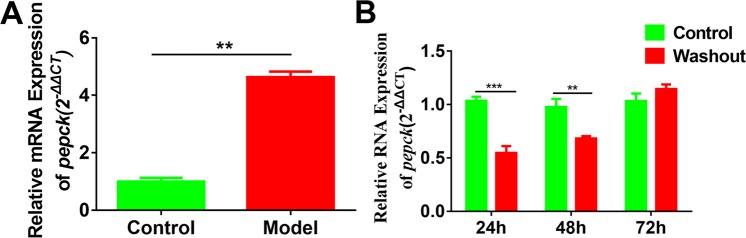
Establishment and stability of diabetic zebrafish model. (**A**) Effect of 100 μM cAMP and 1000 nM DEX treatment for 48 h on the zebrafish fertilized 96 h. The chart showed the relative mRNA expression of PEPCK. **P <0.01; Effect of the washout of 100 μM cAMP and 1000 nM DEX treatment for 48 h on PEPCK gene expression. At least 20 zebrafish larvae were used for each group. **P < 0.01,***P < 0.001.

**Figure 4:**
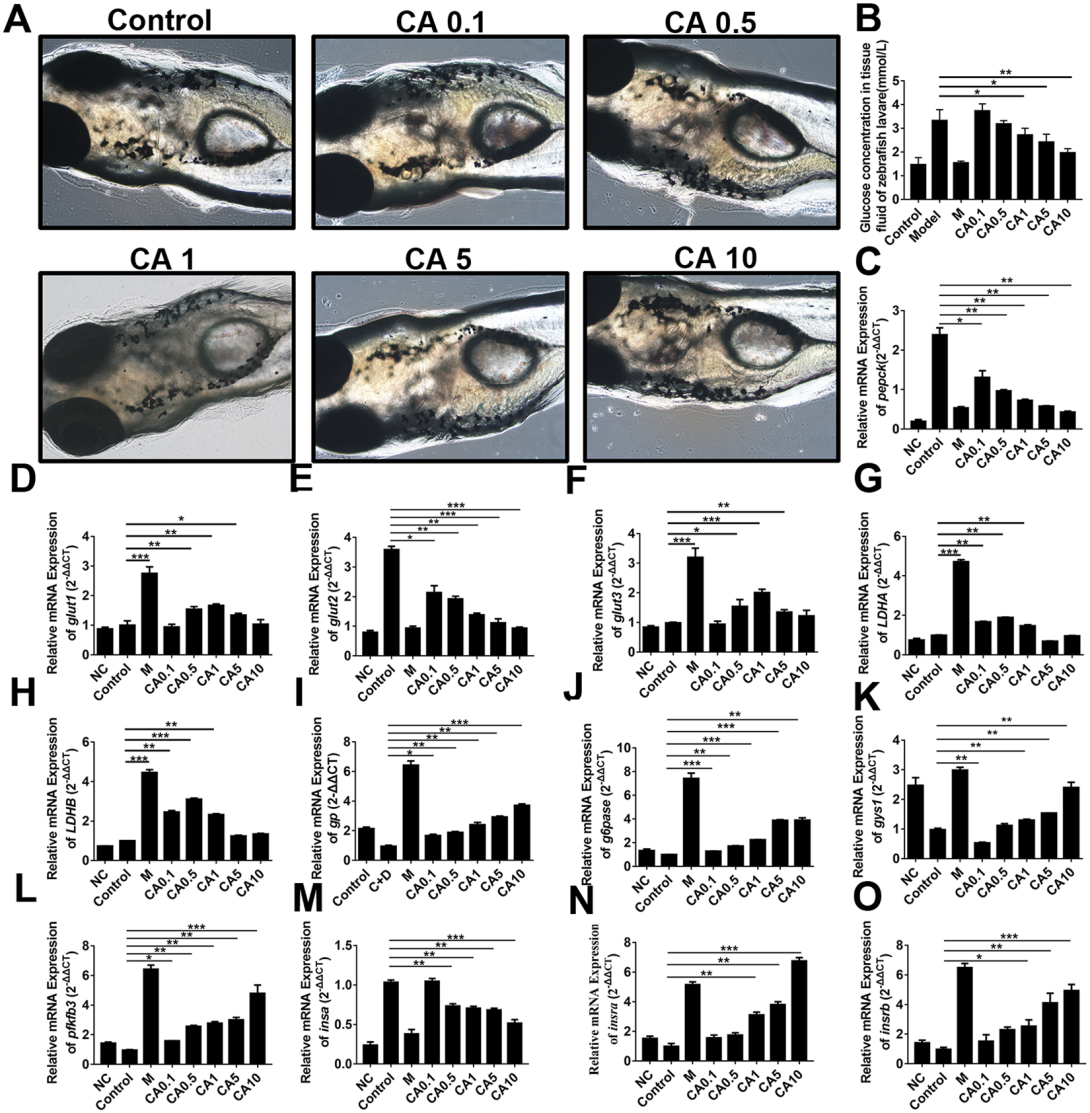
CA reduced glycogen output in zebrafish model. (**A**) The toxicity of CA on zebrafish; (**B**) Glucose concentration in zebrafish tissue fluid treated by different dosages of CA; C-O, PEPCK, GLUT1, GLUT2, GLUT3, LDHA, LDHB, GP, GP6ase, GYS1, PFKFB3, INSα, INSRα and INSRβ mRNA expressions were analyzed by qPCR. *P* < 0.05, **P < 0.01, ***P < 0.0001. NC, negative control; Control, diabetic control; M, metformin.

In our work, zebrafish model was introduced to study the hypoglycemic effect of CA. To establish zebrafish model with PEPCK overexpression, 3 mL of culture medium containing 100 μM cAMP and 1000 nM DEX was added to each well of the treatment group. The results in Fig. 3A indicated that the expression level of *PEPCK* gene was significantly increased about 4.638 times than that of the control group (*P* < 0.01) and glucose level was also upregulated in Fig. 4B. To further confirm the stability of our established diabetic zebrafish model, after incubating 48 h, we replaced the cAMP and DEX solution with normal culture and continued to develop for another 24 h, 48 h, and 72 h, respectively. The result of Fig. 3B presented that *PEPCK* gene expression was conspicuously lower than the corresponding control group at 24 h and 48 h (24 h, *P* < 0.001; 48 h, *P* < 0.01), but *PEPCK* gene expression was slightly higher than the control group without significant difference at 72 h. Therefore, the model’s stability is poor after removing the irritants cAMP and DEX and they are required to exist during incubation periods.

After assessment of Metformin toxicity against zebrafish (Fig. S3, Table S1), the toxicity of CA on zebrafish was firstly determined by morphologic observation and survival statistics. 0.1–10 μM CA have not brought hemolysis and peritoneal hemorrhage in zebrafish larvae (Fig. 4A). After zebrafish larvae cultured in different concentrations of CA for continuous 9 days, only three groups including 10 μM CA at the 7th day, 0.1 μM CA at the 8th day and 10 μM CA at the 9th day, each of which has one zebrafish larva died respectively (Table 3). This result indicated CA within the concentration of 0.1 μM-10 μM has almost non-toxic to zebrafish larvae, which was consistent with our previous T2D HepG2 models.

**Table 3:** Toxicity analysis of CA on zebrafish larvae.

Groups	Zebrafish numbers								
	1d	2d	3d	4d	5d	6d	7d	8d	9d
Control	0	0	0	0	0	0	0	0	0
CA 0.1 μM	0	0	0	0	0	0	0	1	0
CA 0.5 μM	0	0	0	0	0	0	0	0	0
CA 1 μM	0	0	0	0	0	0	0	0	0
CA 5 μM	0	0	0	0	0	0	0	0	0
CA 10 μM	0	0	0	0	0	0	1	0	1

In order to detect whether CA plays a hypoglycemic role by regulating the glycol-metabolism of zebrafish, we firstly detected glucose concentration of zebrafish larvae after treated with different concentrations of corosolic acid. Because the zebrafish juveniles did not bleed, our team added the determination of the zebrafish tissue fluid glucose concentration according to reference^32^. The results indicated that CA reduced glucose level in zebrafish fluid in a dose-dependent manner (Fig. 4B, Table 4).

**Table 4:** Glucose concentration in tissue fluid of zebrafish larvae after treated with different concentrations of corosolic acid (mmol/L).

Group	Control	Model	M	M + CA 0.1 μM	M + CA 0.5 μM	M + CA 1 μM	M + CA 5 μM	M + CA 10 μM
1	1.46 ± 0.30	3.29 ± 0.41	1.52 ± 0.05	3.40 ± 0.43	3.31 ± 0.29	2.38 ± 0.32	2.08 ± 0.50	1.96 ± 0.18

To further discover its underlying mechanism, we analyzed several key enzymes from carbon metabolism in mRNA level including PEPCK, GLUT1, GLUT2, GLUT3, LDHA, LDHB, GP, G6Pase, GYS1 and PFKFB3. The result indicated that CA dramatically downregulated the expression of *pepck* (Fig. 4C), glut1(Fig. 4D), *glut2* (Fig. 4E), glut 3(Fig. 4F), LDHA (Fig. 4G) and LDHB(Fig. 4H), whereas, upregulated the expression of *gp* (Fig. 4I), *g6pase* (Fig. 4J), *gys1* (Fig. 4K) and *pfkfb3* (Fig. 4L) in a dose-dependent manner.

In addition, it’s reported that high stimulation of glucose induced by cAMP and DEX in cell can lead to glucose toxicity and further inhibit the secretion of β cells, the latter of which may induce β cells apoptosis and hyperinsulinemia, and stimulate insulin resistance by reducing the amount of insulin receptor^33^. In our study, we also analyzed the expression of INSα and two of its receptor INSRα and INSRβ. Figure 4M suggested CA significantly downregulated INSα expression in a concentration-dependent manner, which was probably led by the secretion of insulin based on glucose concentration. As shown in Fig. 4N,O, the expressions of two types of insulin receptor were upregulated by CA in a dose-dependent manner(*P* < 0.01).

Moreover, these results indicate cAMP and DEX treatment on zebrafish can establish an insulin resistance model. CA can significantly increase the sensitivity of insulin receptor and improve its effect. Therefore, CA can relieve hyperglycemia, insulin resistance and other symptoms of type 2 diabetes by promoting insulin power.

### CA regulated glucose, lipid metabolism and insulin resistance of STZ-induced type 2 diabetic rats

**Figure 5:**
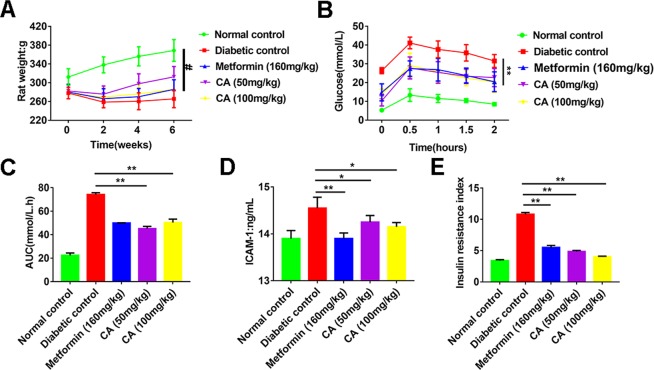
CA regulated glucose, lipid metabolism and insulin resistance of STZ-induced T2D rats. (**A**) Effect of CA on body weight in diabetic rats; Effect of CA on the glucose tolerance (**B**) and AUC(**C**) in diabetic rats; Effect of CA on ICAM-1(**D**) and IRI (**E**) in diabetic rats. The rats were treated with CA (50 or 100 mg/kg) and metformin (160 mg/kg) for 6 weeks. *P < 0.05, **P < 0.01 compared to diabetic groups; ^#^*P* < 0.01 compared to normal control groups.

In this part, a T2D rat’s model was established by both highly fat diet and a low dose of STZ at the same time, with the purpose to discover the effect of CA on hyperglycemia, hyperlipemia and insulin resistance using glucose tolerance and IRI as well-known biomarkers of type 2 diabets^34^. The establishment details about STZ-induced T2D diabetic rats were provided in supporting information (S3) according to previous study^35^. As illustrated in Fig. 5A, with similarly initial body weights, diabetic group’s body weight was remarkably lower than that of the normal control group throughout the entire research (*P* < 0.01). Diabetic rats conducted by CA (50 or 100 mg/kg) showed a tendency to put on weight. CA inhibited the loss of body weight in STZ-induced diabetic rate, and 100 mg/kg CA effects were comparable to metformin group.

In an attempt to measure the consequence of CA on diabetes process caused by high-fat diet and low-dose STZ, we elucidated fasting blood glucose (FBG) levels, glucose tolerance and glycated serum protein (GSP). The six-week administration of CA restrained the estimated increase in the FBG level (Fig. 5B) and augmented the impaired glucose tolerance in T2D rats (Fig. 5C). Moreover, treatment with CA (50 or 100 mg/kg) for 6 weeks declined level of FBG and GSP, and the suppression rates were 86.4%, 91.4%(FBG) and 70.6%, 71.6%(GSP), respectively (Table 5). These results confirmed the antihyperglycemic effect of CA on T2D rats.

**Table 5:** Effect of coroslic acid on biochemical parameters in diabetic rats.

Parameter	Normal control	Diabetic control	Metformin 160 mg/kg	CA 50 mg/kg	CA 100 mg/kg
FBG(mmol/L)	5.51 ± 1.41	26.37 ± 7.82^b^	10.47 ± 5.86^d^	8.25 ± 4.67^d^	7.21 ± 3.11^d^
GSP(mmol/L)	0.61 ± 0.11	1.63 ± 0.44^b^	0.94 ± 0.29^d^	0.91 ± 0.28^d^	0.90 ± 0.32^d^
TC(mmol/L)	2.08 ± 0.41	6.58 ± 1.50	2.59 ± 1.10	2.46 ± 1.15	2.11 ± 0.53
TG(mmol/L)	0.65 ± 0.17	2.71 ± 0.86^b^	1.09 ± 0.67	0.76 ± 0.23	0.81 ± 0.37
HDL-C(mmol/L)	1.71 ± 0.31	0.84 ± 0.26	1.56 ± 0.40	1.55 ± 0.31	1.77 ± 0.33
LDL-C(mmol/L)	0.49 ± 0.23	3.46 ± 0.81	0.66 ± 0.37	0.71 ± 0.29	1.56 ± 0.40
FFA(μmol/L)	0.51 ± 0.06	1.95 ± 0.21^b^	0.53 ± 0.10^d^	1.01 ± 0.15^d^	0.78 ± 0.17^d^
SOD(U/ml)	295.6 ± 45.8	188.7 ± 28.4^b^	291.9 ± 52.2^d^	248.6 ± 39.5^c^	249.8 ± 30.8^d^
MDA(nmol/L)	2.26 ± 0.71	10.83 ± 0.61^b^	4.48 ± 1.78^d^	5.51 ± 2.26^d^	5.12 ± 1.35^d^

The diabetic rats were orally ingested by coroslic acid (50 or 100 mg/kg) and metformin (100 mg/kg) once a day for 6 weeks. The rats of normal control and diabetic control were administrated with vehicle. All above indexes were analyzed after six weeks conducting. Values are recorded by mean ± S.D. for 9 rats in each group. One-way ANOVA test was applied for comparisons between different groups. ^b^P < 0.01 compared with normal control group; ^c^P < 0.05 compared with diabetic control group; ^d^P < 0.01 compared with diabetic control group.

Then, we further illustrated the influences of CA on hyperlipidemia in diabetic rats on assessing the serum levels of HDL-C, LDL-C, TC, TG and FFAs. The results in Table 5 presented that the levels of TC, TG, LDL-C and FFAs were upregulated while the level of HDL-C was downregulated in diabetic rats with significant differences. Administration of CA on diabetic rats remarkably prevented these increases and decreases. These findings show that CA has potential antihyperlipidemic effect in T2D rats.

As we know, the increased level of oxidative stress is the key factor leading to diabetes and its complications. Therefore, in order to exploit the effect of CA on lipid peroxidation, inflammation, and the SOD activity, we also measured MDA and ICAM-1 levels in serum. Compared to normal control rats, MDA and ICAM-1 levels were significantly upregulated, while the SOD activity was remarkably downregulated in diabetic groups (Table 5, Fig. 5D). And rats treated with CA showed decreasing MDA and ICAM-1 levels and increasing SOD activity than diabetic control rats. These results suggest that CA possess crucial antioxidant and anti-inflammatory function in T2D rats.

Insulin resistance is associated with inflammation, which is recognized as potential factor in pathogenesis of T2D^36^. In our study, the IRI (insulin resistance index) in diabetic rats was observably higher than normal control rats, and rats administrated with CA strikingly declined IRI compared to the diabetic rats (Fig. 5E), which show that CA had suppressive effect of insulin resistance.

## Discussion

Type 2 diabetes mellitus (T2DM) as an epidemic disease, associated with increased significant social and financial burden, is a cause of very high morbidity and mortality in the world^2^. It is characterized by abnormal glucose and lipid metabolism due in part to insulin resistance in skeletal muscle, liver and fat, leading to elevated hepatic glucose production, hyperglycemia and hyperlipidemia^37^. Patients with T2DM suffer 2–8 times more deaths than patients with cardiovascular disease^38^. The bioactive products derived from plants, which are with the benefits of availability of materials, affordability, relatively cheap and little or no side effects^39^, are being considered to be a promising source to design effective therapeutic agents for fatal disease including cancer, cardiovascular and diabetes mellitus^40^.

Among numbers of natural products, triterpenoids own various of therapeutic characteristics, containing a hypoglycemic activity^41^. Corosolic acid, a triterpenoid, has been discovered in many Chinese medicinal herbs and been considered as an anti-diabetes agent in food supplementary products^42^. Studies have showed that triterpenoid extract crucially decreased plasma glucose levels in diabetic rats^8^, and corosolic aicd had a lowering effect on post-challenge plasma glucose levels *in vivo* in humans^18^. In addition, many researchers have been trying to find out CA hypoglycemic underlying mechanism. Miura T suggested that the acute hypoglycemic effect of CA was derived, at least in part, from an increase in GLUT4 translocation in mouse muscle, and that CA improved glucose metabolism by reducing insulin resistance^43^. Shi. L demonstrated that CA may exert its antidiabetic effects through enhancing insulin receptor β phosphorylation by inhibiting certain PTPs^20^. Cheng K reported that CA represented a new class of allosteric site inhibitors of glycogen phosphorylase(GP),and their glucose-lowing activity could, at least in part, be due to modulation of glycogen metabolism^44^. In addition, Yamada found that CA increased glucokinase activity without affecting glucose-6-phosphatase activity, suggesting an increase in glycolysis^45,46^. According to the researches to date, no side effects have been reported in animals, nor have adverse events been viewed or reported in controlled human clinical studies. Consider together, it is likely that CA might implement its glucose-lowing effect through multiple targets.

In our study, CA were isolated by semi-preparative high-performance liquid-phase method from *Eriobotrya japonica* leaves of Santan area, the structure and purity of which were confirmed and characterized by NMR, EI-MS spectra and HPLC. Then we invited three different T2D models to systematically research on hypoglycemic effect and potential mechanism of corosolic acid. Phosphoenolpyruvate carboxy kinase (PEPCK) is the rate-limiting enzyme of gluconeogenesis. Enhanced expression of the PEPCK gene in liver is present in most diabetes models and PEPCK overexpression is thought to contribute to the increased hepatic glucose output seen in this disease^47^. A PEPCK gene overexpression HepG2 cell model was established. Different concentrations of CA promoted the consumption of glucose in HepG2 cells, which can improve the degradation of glycogen in cAMP and DEX-induced T2D cell models, inhibited the overexpression of PEPCK.

Zebrafish are complex vertebrates and maintain similarly elaborate mechanisms for activating or mitigating the effects of exogenous chemical substances^48^. It has a short generation time, are highly fertile, and cost little in terms of housing space and daily maintenance owing to their tiny size^49^. Therefore, zebrafish have been used extensively as a model to investigate many vertebrate biological process, including cancer, melanoma, cardiovascular disease, immune system, antioxidative stress and diabetes mellitus^26^. Herein, we firstly established a T2D zebrafish model to evaluate the CA effect on carbon metabolism. This model can not only induce hyperglycemia, but also induce insulin resistance. Our results showed that CA inhibited expression of PEPCK, GLUT1,GLUT2, GLUT3, LDHA, LDHB and INSα, and improve expression levels of GP, GYS1, G6Pase, PFKFB3 and INSR, suggesting the promotion of glycolysis (Fig. S5).

Additionally, T2D rats were treated by high-fat diet and a low dose of STZ. After 6 weeks of continuous gavage administration, the rats suffered from hyperglycemia with higher FBG, GSP, TC, TG, LDL-C and FFA level and lower HDL-C level. Additionally, higher glucose tolerance and insulin resistance index were also shown in diabetic rats, both of which were considered widely as type 2 diabetic markers^34^. Our results revealed that CA alleviated the weight loss of diabetic rats, significantly reduced biochemical parameters including FBG, GSP, TC, TG, LDL-C and FFA level of T2D rats, and improved HDL-C level. And rats treated with CA showed decreasing MDA and ICAM-1 levels and increasing SOD activity than diabetic control rats. Furthermore, rats administrated with CA pronouncedly inhibited IRI compared to the diabetic rats. Taken together, CA can inhibit blood glucose elevation, possess promising antioxidant and anti-inflammatory action, and have an insulin resistance inhibitory effect as well in STZ-induced T2D rats.

## Conclusion

In summary, the results indicate CA extracted from *Eriobotrya japonica* leaves was with high accurate, purity and content. And CA could strongly improve impaired glucose, hyperlipidemia and insulin resistance in T2D models, which were relied on declining the expression of PEPCK and other genes involved in carbon metabolism, T2D related oxidative stress and inflammation. Hence, CA may be a promising agent in the treatment of T2D and more future investigations would be undertaken on its safety and efficacy in clinical trials.

## Experimental Section

### Ethics statement

All animal experiments in this study were conducted in compliance with the Guide for the Care and Use of Laboratory Animals published by the US National Institutes of Health (NIH Publication No. 85-23, revised 1996) and approved by the Animal Care and Use Committee of Nanjing Normal University, China (Permit Number 2090658).

### Chemicals and reagents

Dried *Eriobotrya japonica* leaves were collected from southern part of Anhui province named shexian county. Methanol, ethanol, acetic acid and other all HPLC solvents were bought from Sigma-Aldrich Chemical Co, Ltd. (St. Louis, MO, USA), and all other analytical grade reagents were obtained from Aladdin (Shanghai, China). Corosolic acid (CA) standards (HPLC ≥ 95%) were purchased from Jiangsu Institute for Food and Drug Control. Metformin HCL, DEPC, Dexamethasone, Adenyl-3′, 5′-cyclic phosphate, STZ, Sodium citrate, Citric acid and Glucose were purchased from Sigma Aldrich.

Dulbecco’s Modified Eagle’s Medium (DMEM) and fetal bovine serum (FBS) were purchased from Gibico (Thermo Fisher Scientific, Grand Island, USA). Cell counting kit-8(CCK-8), RNA isolater^TM^ Total RNA Extraction Reagent, HiScript^TM^ II Q RT SuperMix for qPCR and AceQ qPCR SYBR Green Master Mix (High ROX) were purchased from Vazyme Co. Ltd. (Nanjing, Jiangsu, China). Intercellular Adhesion Molecule-1 (ICAM-1) ELISA Kit and Insulin ELISA Kit were purchased from Dakewe Biotech Co., Ltd (Shanghai, China). Glucose assay kit, Muscle/liver glycogen test kit, glycated serum protein (GSP), FFAs, malondialdehyde (MDA) and superoxide dismutase (SOD) were purchased from Nanjing Jiancheng Bioengineering Institute (Nanjing, China). The kits for measurement of total cholesterol (TC), triglyceride (TG), high density lipoprotein-cholesterol (HDL-C), low density lipoprotein-cholesterol (LDL-C) were purchased from Sigma-Aldrich Chemical Co, Ltd. (St. Louis, MO, USA).

Preparation of 4 mM dexamethasone (DEX) mother liquor: Weigh 0.0167 g of DEX powder, dissolve it with appropriate amount of ultrapure water, sonicate for 20 min, dilute to 10 ml, configure into 4000 μM DEX mother liquor, store at −20 °C, and leave for use.

Preparation of 5 mM adenyl-3′,5′-cyclic phosphate (cAMP) mother liquor: Weigh 0.0165 g cAMP powder, add appropriate amount of ultrapure water to dissolve, sonicate for 20 min, dilute to 10 ml, configure into 5000 μM cAMP mother liquor, place Store at −20 °C and leave for spare.

Preparation of 10 × E3 zebrafish embryo culture solution: Weigh NaCl: 1462.5 mg, KCl: 63.325 mg, CaCl_2_: 242.583 mg, MgSO_4_: 198 mg, add appropriate amount of mono-distilled water to dissolve, sonicate for 20 min, after all dissolved, transfer to 500 mL volumetric flask Medium, constant volume, the prepared mother liquid is stored in a refrigerator at 4 °C, and diluted 10 times with single distilled water.Preparation of a molding agent: A solution having a final concentration of 100 *μ*M (cAMP) and 1 *μ*M (DEX) was prepared using 1 × E3.

Adult *Tuebingen* zebrafish were brought from Model Animal Research Center of Nanjing University and Male Sprague-Dawley rats (180–200 g) were purchased from Qing Longshan Animal Breeding Farm in Nanjing. Animal quality certificate number: SYXK (Su) 2014-0052.

### Cell culture, animals and treatments

Human liver hepatocellular carcinoma cells (HepG2) were kept by our own lab and cultured in DMEM supplemented with 10% FBS in a humidified atmosphere containing 5% CO_2_ at 37 °C.

The wild type zebrafish was reared with filtered tap water, with a photoperiod of 14 h (daylight)/10 h (dark night) and a temperature of 28 ± 1 °C. Zebrafish are fed twice a week with dried fish and brine shrimp^50^. Eggs were collected from natural spawning that was induced in the morning by turning on the light. The eggs were obtained within 30 min after fertilization and incubated in double distilled water until the desired developmental age.

SD Rats were fed with normal diets (21.5% protein, 6.5% calories, 4.1% fiber, 8% ash, and 2.8% minerals) and were allowed to eat freely in the laboratory. The plastic cage was filled with tap water (four to five per cage), the temperature was controlled at (22 ± 2 °C), relative humidity (50% to 60%), and a 12 h light/dark cycle was maintained. The rats were domesticated for a week.

After continuous gavage for six weeks, rats in each group were anesthetized with sodium pentobarbital (60 mg/Kg) intraperitoneally and euthanized by cervical dislocation. Blood was taken from the femoral artery and placed in a centrifuge tube coated with EDTA. The sample was centrifuged at 3000 rpm for 10 minutes at 4 °C. The serum was collected and stored at −20 °C until use.

### Cytotoxicity assay of HepG2 cells ***in vitro***

5 × 10^4^ cells/mL HepG2 cells were added to 96-well cell culture plates at 100 μL per well. They were incubated in a 37 °C, 5% CO_2_ incubator for different times. Cytotoxicity assay was measured by CCK8 kit according to the manufacturer’s protocols.

### Cellular glucose consumption assay

Cells were randomly divided into different group containing low glucose DMEM medium. After 12 hours, 24 hours, or 36 hours incubation, glucose assay kit was used to measure the glucose consumption. Add 10 μL CCK8 cross-shaking 96-well plate to each well. After incubation for 1 h in a cell culture incubator, the absorbance was measured at 450 nm, and the glucose consumption per well was calculated by glucose content divided corresponding OD value of CCK8 to obtain the unit glucose consumption per well. Three replicated wells were used in each group and the experiment was repeated three times.

### Cell glycogen accumulation assay

HepG2 cells were kept with serum-free high-glucose DMEM culture medium and starved for 12 hours. The cells were randomly divided into different groups and cultured in low glucose medium for another 12 hours. Then the cells were collected and washed twice with 1 mL of 1X PBS. The glycogen content of cells in each experimental treatment group was subsequently determined following a liver glycogen assay kit.

### Glucose concentration detection in tissue fluid of zebrafish larvae

Whole body glucose was determined by using an Amplex Red glucose assay kit (Life Technologies) according to the instructions.

### Establishment of diabetic zebrafish model

After 96 hours of fertilization, zebrafish juveniles which have fully developed organs including the liver and pancreas were sowed in six-well cell culture plates, 20 zebrafish juveniles were added to each well, and 3 mL cultures were added to the blank group. 3 mL of culture medium containing 100 μM cAMP and 1000 nM DEX was added to each well of the treatment group. After incubated for 48 h, total mRNA was extracted using the total RNA extraction kit for RT-qPCR, and the expression level of PEPCK gene was detected.

### Zebrafish toxicity assay

The fertilized fish eggs were collected by plastic pipettes, sown in 24 well plates, 6 per well, and randomly divided into groups with different CA concentrations (0.1, 0.5, 1, 5, 10 μM). Toxicity test was further determined by the observation of the survival of zebrafish juveniles and the presence of hydrocele every day. Fish were not fed and dead fish were immediately removed from the wells.

### Blood glucose detection assay in rats

The rats were abstained from food except water for 12 h. At 0 and 120 min, the venous plexus blood was taken for blood glucose determination. The levels of glucose in rat serum were measured according to the test methods specified in the kit’s instructions.

1. The area under the blood glucose curve (AUC) is calculated as follows:
  1 $${{\rm{AUC}}}_{{\rm{glu}}}={\rm{0.5}}\times (\mathrm{Bg0}+\mathrm{Bg5})/2+{\rm{0.5}}\times ({\rm{Bg0}}\mathrm{.5}+\mathrm{Bg1})/2+{\rm{0.5}}\times (\mathrm{Bg1}+\mathrm{Bg1}{\rm{.5}})/2+{\rm{05}}\times ({\rm{Bg1}}\mathrm{.5}+\mathrm{Bg2})/2$$
  Bg0, Bg0.5, Bg1, Bg1.5, and Bg2 correspond to glucose concentrations after 0, 0.5, 1, 1.5, and 2 h glucose loading, respectively.
2. The inhibition rate of fasting blood glucose is calculated according to the following formula:
  2 $${\rm{Inhibition}}\,\mathrm{rate}( \% )=(\mathrm{C1}\,-\,\mathrm{C2})/(\mathrm{C1}\,-\,\mathrm{C0})\times {\rm{100}}$$
  C0: fasting blood glucose in the normal control group,
  C1: fasting blood glucose in the diabetic control group,
  C2: fasting blood glucose in the administration group.
3. The insulin resistance index (IRI) is calculated according to the following formula:

3 $${\rm{IRI}}={\rm{FBG}}\times \mathrm{FBI}/\mathrm{22}{\rm{.5}}$$

FBG: fasting blood glucose; FBI: fasting blood insulin levels.

### Oral glucose tolerance test in rats

The oral glucose tolerance test was performed in the fifth week. After continuous administration of corosolic acid for 5 weeks, rats fasted for 12 h. Blood was then taken from the venous plexus of the rat’s eyelid to measure the blood glucose level of fasting blood glucose for 0 min. Rats received oral glucose 2.0 g/kg. Blood samples were collected 30 min, 60 min, 90 min, and 120 min after glucose loading. Blood glucose levels were measured with a kit, and changes in OGTT before and after treatment were observed.

### RNA extraction and quantitative real time PCR (qRT-PCR)

After different treatment, total RNA was extracted from cells using the RNA isolater^TM^ Total RNA Extraction Reagent (Vazyme Co. Ltd.). RNA concentration was determined using a nucleic acid protein assay, and cDNA was synthesized from the total RNA using the HiScript^TM^ II Q RT SuperMix for qPCR kit (Vazyme Co.Ltd.) according to the manufacturer’s instructions. To assess the expression of genes in each treatment group, the cDNA was used as the template for real-time PCR using AceQ qPCR SYBR Green Master Mix (High ROX) kit (Vazyme Co. Ltd.). All primers were listed in Table 6 ^27^.

**Table 6:** Danio rerio primers for RT-PCR assays.

Name	Accession	Direction	Primer sequence (5′-3′)
**Target gene**			
PEPCK	NM_002591.3	Forward	AAAACGGCCTGAACCTCTCG
		Reverse	ACACAGCTCAGCGTTATTCTC
INS	NM_131056.1	Forward	AGTGTAAGCACTAACCCAGGCACA
		Reverse	TGCAAAGTCAGCCACCTCAGTTTC
INSRa	NM_001142672.1	Forward	CAACATGCCCCCTCACCACT
		Reverse	CGACACACATGTTGTTGTG
INSRb	NM_001123229.1	Forward	GACTGATTACTATCGCAAGGG
		Reverse	TCCAGGTATCCTCCGTCCAT
GLUT1	NM_001039808.1	Forward	TCCCATGGTTCATTGTGGCA
		Reverse	GCCGGTCAGTTCCTCAACAT
GLUT2	NM_001042721.1	Forward	GGGATACAGCTTGGGCGTCATC
		Reverse	GGACAACATGCCTCCGACAGAGA
GLUT3	NM_006931.3	Forward	ACTTGCTGCTGAGAAGGACAT
		Reverse	GGGTGACCTTCTGTGTCCCC
LDHA	NM_131246.1	Forward	TTCCTCACATCACCAGTGCG
		Reverse	GAGGCCATCTTCAACTGTGG
LDHB	NM_001174097.2	Forward	AGCGGAGAGACTTGTTGCATT
		Reverse	CAGCCAGAGACTTTCCCAGAA
GP	NM_001008538.1	Forward	CTTCCCTGATAAGGTTGCCA
		Reverse	TCCACAAATATCCGCATCAA
GYS1	NM_201180.2	Forward	CTCTCCTCAACTTCTCCTCTC
		Reverse	CTCGGCTGGTGTGTATCC
G6Pase	NM_001163806.1	Forward	CCACACCGTTCGCAAGTC
		Reverse	CCACACCGTTCGCAAGTC
PFKFB3	NM_213397.1	Forward	TTCGCAGGTGCTCTTGCTA
		Reverse	GTTCGTACGGCACATTGAGA
β-actin	NM_131031.1	Forward	CGAGCAGGAGATGGGAACC
		Reverse	CAACGGAAACGCTCATTGC

### Statistical analysis

All the experimental results were triply performed and analyzed by GraphPad Prism 7 software (GraphPad Software, Inc, La Jolla, CA, USA). The data are presented as mean ± SEM. The statistical analyses were assessed by Student’s t-test. P-value < 0.05 was considered significantly difference.

## Supplementary information

Dataset1
