# Supplementary material for: Corosolic acid isolated from Eriobotrya japonica leaves reduces glucose level in human hepatocellular carcinoma cells, zebrafish and rats
Source: Sci Rep. 2019 Mar 13;9:4388. doi: 10.1038/s41598-019-40934-7 (PMC6416347; doi:10.1038/s41598-019-40934-7)
Supplement: Supplementary file 1 — Dataset1 [file 41598_2019_40934_MOESM1_ESM.pdf]

Supporting information

Corosolic acid isolated from *Eriobotrya japonica* leaves reduces glucose level in human hepatocellular carcinoma cells, zebrafish and rats.

Shuwen Xu[1 2]#, Gang Wang[1]#, Wei Peng[1], Yandi Xu[1], Yu Zhang[1], Ying Ge[1], Yue Jing[3]* and Zhunan Gong[3]*

[1] Center for New Drug Research and Development, College of Life Science, Nanjing Normal University, Nanjing 210023, People's Republic of China

[2] Department of Scientific Research Management, Anhui Academy of Science and Technology, Hefei 230088, People's Republic of China

[3] Central Laboratory of Stomatology, Nanjing Stomatological Hospital, Medical School of Nanjing University, Nanjing 210008, People's Republic of China

Address correspondence to Professor Zhunan Gong, Room 607, Life Science Building, No. 1, Wenyuan Road, Nanjing Normal Univeristy, Nanjing, Jiangsu Province, China. Zip code: 210023.Tel:+86-25-85891736; Fax:+86-25-85891736; E-mail: gongzhunan@njnu. edu.cn;

Ph.D Yue Jing, No.30 Zhongyang Road, Central Laboratory of Stomatology, Nanjing Stomatological Hospital, Medical School of Nanjing University, Nanjing, Jiangsu Province, China. Zip code:210008. Tel.:+86-25-83620140; Fax:+86-25-8589140;

E-mail: jingyueshadow@126.com.

* Author to whom correspondence should be addressed.

#These authors contributed equally to this work.

Table of contents	Pages
HPLC methodology	S1
Type 2 diabetes mellitus in HepG2 cell	S1-S2
Toxicity of metformin	S3
STZ-induced Type 2 diabetic rat	S3-S4
Toxic and nontoxic zebrafish	S4
Hypothetic mechanism	S4

1. HPLC methodology to determine the content of CA in *Eriobotrya Japonica* leaves

1.1 Chromatographic condition

Samples were analyzed by a Phenomenex-C_{18}(250 mm×4.6 mm, 5μm), KNAUER PumpK-1800 UVK02 system at 25℃ using mobile phase (methanol:1%phosphoric acid=88:12) at 0.8mL/min flow speed. The sample injection volume was 20μL and the detection wavelength was 215nm.

1.2 Solution preparation

Standard solution: CA standard was dried at 105℃, accurately weighed 10.0mg out and dissolved with methanol in 10.0mL volumetric flask.

Test solutions: *Eriobotrya japonica* leaves were dried, smashed and sifted through 60-mesh sieve. 2.0g powders were purified triply with 20mL 90% ethanol at 90℃ for three hours. After cooling down, combine three extraction and metered to 100mL volume with 90% ethanol. Then filtrated with distilled water twice and dissolved the precipitate with methanol to 100mL.

1.3 Standard curve

CA standard solutions were injected in 1, 2, 3, 4, 5, and 6 μL, respectively, and the peak area was determined according to determination of chromatographic conditions. With the peak area as the ordinate and the reference sample volume as the abscissa, the standard curve was plotted: Y=335.538X-1.371 (r^2=0.99989). We can see that under the analyze chromatographic conditions, the CA samples were injected at 1.0 to 6.0 μL with good linearity.

1.4 Precision experiments

5 μL CA control solutions were injected 6 times. Peak area was determined and RSD was 1.5%.

1.5 Stability and repeatability experiments

Take the sample to prepare 6 parallel samples as the test solution and inject 10 μL. The mass fraction of the triterpene acid is CA: 8.56 mg/g and RSD was 2.5% with excellent stability.

1.6 Recovery ratio experiments

The sample recovery ratio was determined by taking 1.0g each from 5 content-known samples. Add 5.0 mg CA and prepare the test solution. The average recovery rate of CA is 99.0%. RSD was 0.36%.

2. Type 2 diabetes model in HepG2 cell

2.1 HepG2 cell model establishment

HepG2 cells were cultured with serum free low sugar DMEM medium containing different concentrations of cAMP (adenosine-3',5'-cyclic phosphate) and DEX (dexamethasone) for 12h, 24h and 36 h, respectively. Then glucose consumptions of different mediums were measured according to the instructions of the kit. The results showed that at 12 hours (Figure S1A), except for 500μM cAMP-treated group, the unit glucose consumptions of other cAMP and DEX-treated

groups were decreased, especially the groups of 100μM cAMP +1000nM DEX and 500μM cAMP +1000 nM DEX decreased to 76% and 81% comparing to control group (*P*<0.001). However, there were no significant difference between the cAMP and DEX-treated groups with control group at 24h and 36h (Figure S1B & S1C). Therefore, 100μM cAMP +1000nM DEX was the best condition for type 2 diabetic cell models.

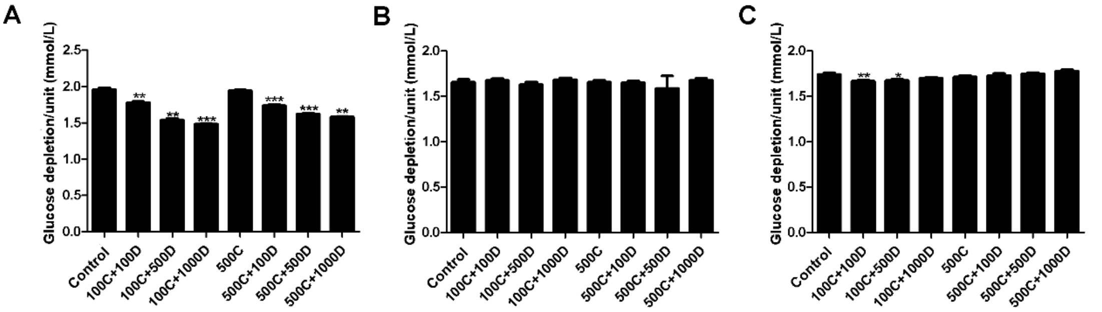

Figure S1. Establishment of type 2 diabetes model in HepG2 cell. HepG2 cells were treated with cAMP and DEX in different concentrations and times (A, 12h; B, 24h; C, 36h), and glucose depletion per unit was measured by glucose assay kit. All culture mediums were serum free and low sugar. 100C+100D, 100μM cAMP +100nM DEX; 100C+500D, 100μM cAMP +500nM DEX; 100C+1000D, 100μM cAMP +1000nM DEX; 500C, 500μM cAMP; 500C+100D, 500μM cAMP +100nM DEX; 500C+500D, 500μM cAMP +500nM DEX; 500C+1000D, 500μM cAMP +1000nM DEX; "*" indicated that the unit glucose consumption of the treated group was significantly different from the control group, **P*<0.05, ***P*<0.01, ****P*<0.001.

2.2 Stability of HepG2 cell model

To ensure the stability of our established model, HepG2 cells were starved with serum free high sugar DMEM for 12h after treated with different concentrations of cAMP and DEX for 12h. Then cells were continuous cultured with low sugar DMEM for 12h, 24h and 36h. The results in Figure S2 pointed out that the decrease of glucose depletion disappeared after removal of cAMP and DEX. As a result, the stability of the model could not be separated from the stimulation of cAMP and DEX.

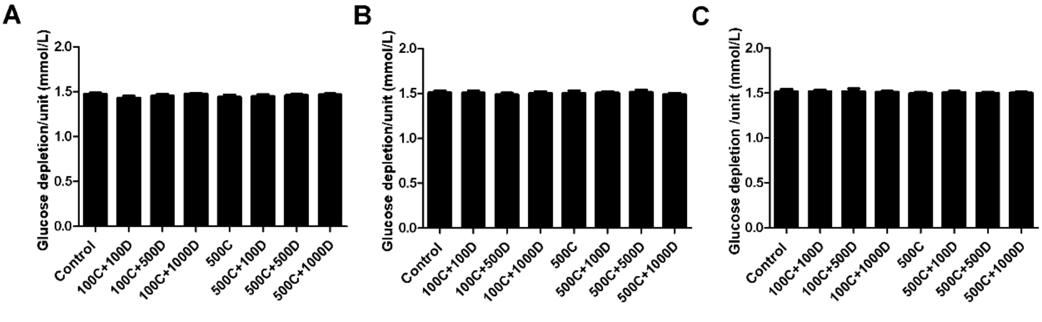

Figure S2. The stability of type 2 diabetes model in HepG2 cell. After removing cAMP and DEX, cells were cultured for another 12h(A), 24h(B) and 36h(C). Glucose depletion disappeared and no significant differences were found among different groups. 100C+100D, 100μM cAMP +100nM DEX; 100C+500D, 100μM cAMP +500nM DEX; 100C+1000D, 100μM cAMP +1000nM DEX; 500C, 500μM cAMP; 500C+100D, 500μM cAMP +100nM DEX; 500C+500D, 500μM cAMP +500nM DEX; 500C+1000D, 500μM cAMP +1000nM DEX.

3. Toxicity of metformin

To ensure the toxicity of metformin against zebrafish, we treated different concentrations of metformin for different times. In our work, 10μM Metformin was used as positive control. From toxicity of metformin, it is almost no toxic of 10μM Metformin to zebrafish in 72hpf.

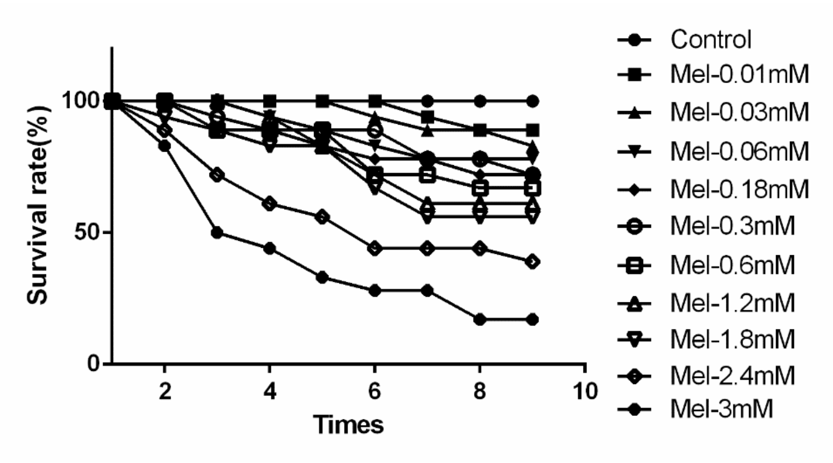

Figure S3. Toxicity of Metformin against zebrafish

Table S1 Toxicity analysis of Mel on zebrafish larvae

Groups	Zebrafish mortality（%）								
	1d	2d	3d	4d	5d	6d	7d	8d	9d
Control	0	0	0	0	0	0	0	0	0
Mel 0.01mM	0	0	0	0	0	0	0	0	0
Mel 0.03mM	0	0	0	0	0	6	11	11	17
Mel 0.06mM	0	0	0	6	11	17	22	22	22
Mel 0.18mM	0	0	11	11	17	22	22	28	28
Mel 0.3mM	0	0	6	11	11	11	22	22	28
Mel 0.6mM	0	0	11	11	11	28	28	33	33
Mel 1.2mM	0	0	0	6	17	28	39	39	39
Mel 1.8mM	0	6	11	17	17	33	44	44	44
Mel 2.4mM	0	11	28	39	44	56	56	56	61
Mel 3mM	0	17	50	56	67	72	72	83	83

4. STZ-induced Type 2 diabetic rat

After a week of domestication, rats were fed with high glucose diet (12.5% lard, 15% sucrose, 5% egg yolk powder, 1% cholesterol, 0.5% sodium cholate and 66% standard laboratory) for four weeks. The diabetic model was induced by intraperitoneal injection of STZ 35mg/kg (dissolved by 0.1M pH 4.5 citric acid buffer) after fasting for 12hours. One week after injection, rats fasted for 12 hours and were given 2g/kg glucose. Blood samples were taken from venous plexus to determine the glucose level at 0 min and 120 min separately. The blood glucose levels in 0 min ≥7.8mmol/L and 120min ≥11.1mmol/L for type 2 diabetic rats were confirmed as successful rats.

The control group was fed with diet containing 21.5% protein, 6.5% heat, 4.1% fiber, 8% ash and 2.8% minerals and then injected by 0.1M pH 4.5 citric acid buffer. After fasting for 12 hours, blood samples were collected from venous plexus at 0 min and 120 min. Rats with normal blood glucose were identified in the range of 4.4 to 7.9 mmol/L and served as the normal control group.

5. Toxic and nontoxic zebrafish

From the figure S4 below, toxic zebrafish shows characteristics like bleed, pericardial edema, yolk sac swelling.

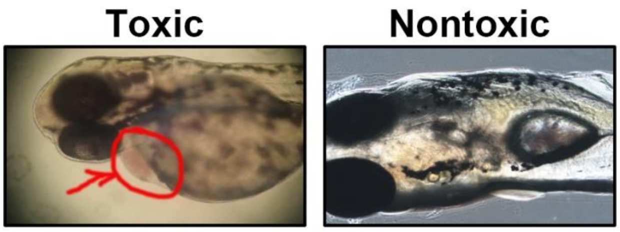

Figure S4. Toxic and nontoxic zebrafish.

6. Hypothetic signal pathway

In the revised manuscript, we have added the expression of GLUT1, GLUT3, LDHA, and LDHB regulated by corosolic acid in figure 4D, F, G and H. And the results showed CA reduced GLUT1, GLUT3, LDHA and LDHB. Basing on the expression of GLUT1, GLUT3, LDHA, LDHB GLUT2, GP, G6Pase, GYS1, PFKFB3, INSα, INSRα and INSRβ regulated by CA, we conferred Warburg and AMPK pathway might be involved by CA-reducing glucose effects (Shown in Fig S5)

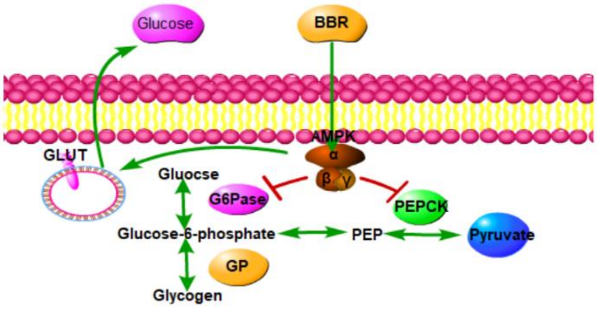

Figure S5. The mechanism of CA reducing glucose
